# Better, a surgeon’s notes on performance: Atul Gawande: Profile Books, London, 2008

**DOI:** 10.1007/s40037-012-0003-6

**Published:** 2012-02-08

**Authors:** Fedde Scheele

**Affiliations:** Department of Obstetrics and Gynaecology, VU University Medical Centre, Amsterdam, The Netherlands

A dear colleague gave me the book ‘Better, a surgeon’s notes on performance’ by Atul Gawande as a present. He seemed surprised that I had not read any books by this writer. Being very much involved in training in generic competencies and being an enthusiastic storyteller and listener, this book was the perfect gift for me. The book is about generic competencies told from the heart of a surgeon. Atul observes the world around him and discusses questions about medical performance. Let me quote from the book:

‘As a doctor you go into this work thinking it is all a matter of canny diagnosis, technical prowess, and some ability to empathize with people. But it is not, you soon find out. In medicine, as in any profession, we must grapple with systems, people—and our own shortcomings, as well. We face obstacles of seemingly unending variety. Yet somehow we must advance, we must refine, we must improve. How we have and how we do is my subject here. …’.

The book is divided into three sections. The first section is about diligence, the second about to do right by people and the third about ingenuity. Atul is a superb storyteller. When you start reading this book, he takes you straight into the fascinating world of medicine. The stories deal with very different subjects, ranging from medical care in the war in Iraq to washing hands in your own hospital. He combines a positivistic view of medical progress with a very intelligent analysis of what opportunities we have to improve our performance. The story about bench-marking the results of care for patients with cystic fibrosis and the positive effect in all of the centres striving for excellence is an excellent example of a lesson that should be taught to everyone working in patient care.

It is hard to read this book without strong emotions. We are all part of the system. We are all faced with the huge challenge of improving the performance of health care workers. We are all familiar with the characteristics of the system we work in and doubt whether we will ever be able to create change and improvement. Atul Gawande’s stories are lessons for life that will give you strength and courage to keep working towards a better health care system. They teach you to stop complaining, to observe, to be creative and to embrace change.

This book, and the other books by Atul Gawande, ‘Complications’ and ‘The Checklist Manifesto’, should be compulsory reading for every doctor. These books are important to discuss with our students and our postgraduate trainees. They will enjoy reading the brilliant stories, but more importantly, they will be offered a glance into the health care of the future, which will be in their hands.

Fedde Scheele (I am now a great fan of Atul Gawande, Thanks!)

